# Molecular Characterization and Functional Analysis of the Dipeptidyl Peptidase IV from Venom of the Ectoparasitoid *Scleroderma guani*

**DOI:** 10.3390/toxins15050311

**Published:** 2023-04-27

**Authors:** Chaoyan Wu, Cheng Yang, Yuqin Wang, Jun Wang, Jiaying Zhu

**Affiliations:** 1 Key Laboratory of Forest Disaster Warning and Control of Yunnan Province, College of Biodiversity Conservation, Southwest Forestry University, Kunming 650224, China; 2 Key Laboratory for Forest Resources Conservation and Utilization in the Southwest Mountains of China, Ministry of Education, College of Biodiversity Conservation, Southwest Forestry University, Kunming 650224, China

**Keywords:** dipeptidyl peptidase IV, venom, parasitoid, transcriptome, gene expression

## Abstract

Dipeptidyl peptidase IV (DPPIV) is a proline-specific serine peptidase that remains poorly investigated in terms of venom composition. Here, we describe the molecular characteristics and possible functions of DPPIV as a major venom component of the ant-like bethylid ectoparasitoid, *Scleroderma guani*, named SgVnDPPIV. The SgVnDPPIV gene was cloned, which encodes a protein with the conserved catalytic triads and substrate binding sites of mammalian DPPIV. This venom gene is highly expressed in the venom apparatus. Recombinant SgVnDPPIV, produced in Sf9 cells using the baculovirus expression system, has high enzymatic activity, which can be efficiently inhibited by vildagliptin and sitagliptin. Functional analysis revealed that SgVnDPPIV affects genes related to detoxification, lipid synthesis and metabolism, response to stimuli, and ion exchange in pupae of *Tenebrio molitor*, an envenomated host of *S. guani*. The present work contributes towards understanding the role of venom DPPIV involved in the interaction between parasitoid wasp and its host.

## 1. Introduction

Dipeptidyl peptidase IV (DPPIV, EC 3.4.14.5) belongs to the subfamily S9B of serine peptidases from peptidase clan SC in the MEROPS database (https://www.ebi.ac.uk/merops/ (accessed on 6 January 2023) [1]. As an aminopeptidase, it preferentially cleaves dipeptides from peptides with a penultimate proline or alanine in the P1 position, such as chemokines, neuropeptides, and peptide hormones [2]. This enzyme is found in a wide range of different living organisms, including mammals, insects, plants, and microorganisms, which has been mostly and best studied in humans [3]. Human DPPIV is widely distributed in cells and tissues to act in various physiological and pathological processes, ranging from glucose homeostasis, immunoregulation, and inflammation to tumorigenesis, through an anchored transmembrane molecule or a soluble circulating enzyme [4,5]. The prominent role of this enzyme is to rapidly inactivate the incretin peptide hormones, glucagon-like peptide-1 and glucose-dependent insulinotropic polypeptide, that are involved in regulating blood glucose levels [6]. Thus, it provides as an ideal pharmacological target to develop drugs for the treatment of type 2 diabetes by potentiating glucose-dependent insulin secretion [7,8].

Compared with mammalian DPPIV, little is known about insect DPPIV, with information derived from limited studies conducted on a few species, including blue blowfly *Calliphora vicina* [9], cockroach *Leucophaea maderae* [10], fruit fly *Drosophila melanogaster* [11], yellow mealworm *Tenebrio molitor* [3], honey bee *Apis mellifera* [12], and several wasps [12,13,14,15,16,17]. Insect DPPIV was first partially purified from *C. vicina*, which has been shown to be able to degrade the ecdysiostatic peptide, i.e., trypsin-modulating oostatic factor, of the grey flesh fly *Neobellieria bullata* [9]. Partly purified DPPIV activity was characterized from the brain and midgut of *L. maderae*, and is thought to participate in the inactivation of tachykinin-related peptides [10]. A gene (*omega*) encoding DPPIV was cloned from *D. melanogaster* [11]. However, the functions of DPPIV from these insects are not explored. It was found that a soluble DPPIV from the anterior midgut of *T. molitor* larvae can efficiently hydrolyze gliadins, the main dietary protein of the insect, showing that it has a digestive function [3]. Interestingly, DPPIV is the major component in the venom of hymenopterans. This enzyme from the hornets *Vespa basalis* and *V. magnifica*, has been considered as the putative enzyme used to process toxins, including mastoparan B and vespid chemotactic peptide, with antimicrobial activities [13,14,17,18]. It is a major venom allergen of *A*. *mellifera*, *Polistes dominula,* and *V*. *velutina nigrithorax* [12,15,16].

Parasitoids likely comprise the largest group of venomous organisms, and are one of the most diverse groups of Hymenoptera [19]. Their venoms comprise a complex cocktail of proteinaceous and non-proteinaceous components with diverse biological functions that facilitate the successful development of its offspring either inside or outside the host [20,21]. Enzymatic proteins, such as serine proteinase, esterase, and metalloprotease, constitute the most abundant venom components of parasitoids [22,23,24]. Of these, DPPIV has been commonly found [25,26]. Unfortunately, the molecular characteristics and functions of DPPIV as a constituent of parasitoid venoms are still unknown.

The ant-like bethylid wasp, *Scleroderma guani*, is a valuable biological control agent that can parasitize the larvae or pupae of many woodboring beetles [27], and has been successfully used to control the Japanese pine sawyer, a notorious forest pest in Asia known as an efficient vector of the destructive pine wilt disease [28]. A DPPIV (SgVnDPPIV) was identified as a major venom component of this ectoparasitoid [29]. We here describe the molecular characterization and functional analysis of SgVnDPPIV.

## 2. Results

### 2.1. Molecular Characteristics of SgVnDPPIV

**Figure 1:**
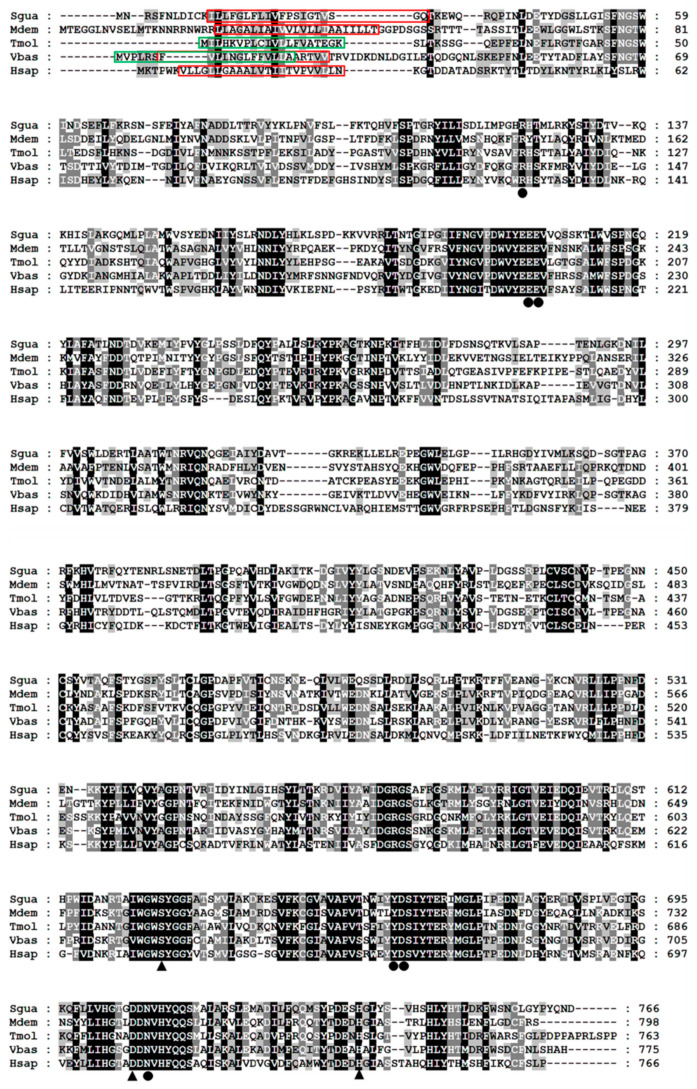
Multiple alignment of SgVnDPPIV and DPPIVs from other insects and humans. Sgua, *Scleroderma guani*; Tmol, *Tenebrio molitor* (CAH1368919); Vbas, *Vespa basalis* (ABG57265); Mdem, *Microplitis demolitor* (XP_008553451); Hsap, *Homo sapiens* (P27487). The transmembrane domains of *S. guani*, *V. basalis*, *M. demolitor*, and *H. sapiens* DPPIVs are marked in red boxes. The signal peptides of *T. molitor* and *V. basalis* DPPIVs are indicated in green boxes. The catalytic triads and substrate binding sites are indicated with triangles and solid circles, respectively. The identical and highly conserved residues are shaded in black and grey, respectively.

**Figure 2:**
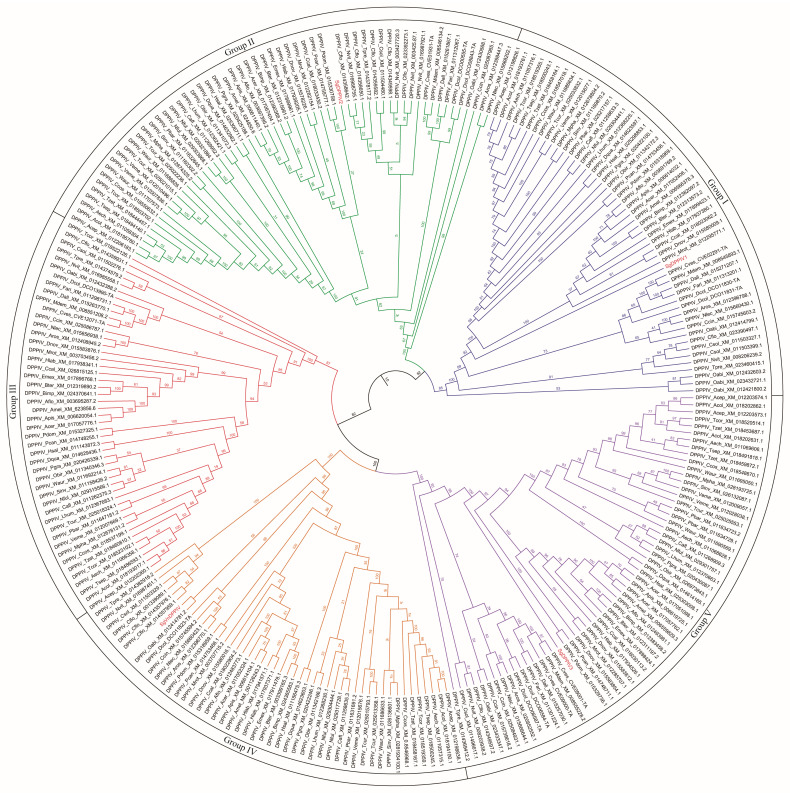
Phylogenetic tree of DPPIVs from various hymenopteran species. Amino acid sequences of DPPIVs used to construct the phylogenetic tree are listed in Table S2.

The cloned SgVnDPPIV gene sequence is 2722 bp in length, containing an open reading frame (ORF) of 2301 bp (Figure S1). The predicted protein contains 767 amino acids, with a calculated molecular weight of 87.30 kDa and isoelectric point of 6.34. There is no signal peptide predicted in the deduced amino acid sequence. SgVnDPPIV shares 47%, 38%, 34%, and 31% identity with DPPIVs of *V. basalis*, *T. molitor*, *Microplitis demolitor*, and *Homo sapiens*, respectively (Figure 1). The catalytic triads and substrate binding sites of mammalian DPPIV are conserved in SgVnDPPIV. Genes encoding DPPIVs in the genomes of different hymenopteran species vary from 4 to 11 (Tables S1 and S2). In addition to the SgVnDPPIV gene, there are three DPPIV genes identified in the genome of *S. guani.* Phylogenetic analysis revealed that hymenopteran DPPIVs can be divided into five groups (Figure 2). SgVnDPPIV and the other three DPPIVs of *S. guani* are grouped into different clades.

### 2.2. Gene Expression Pattern of SgVnDPPIV

**Figure 3:**
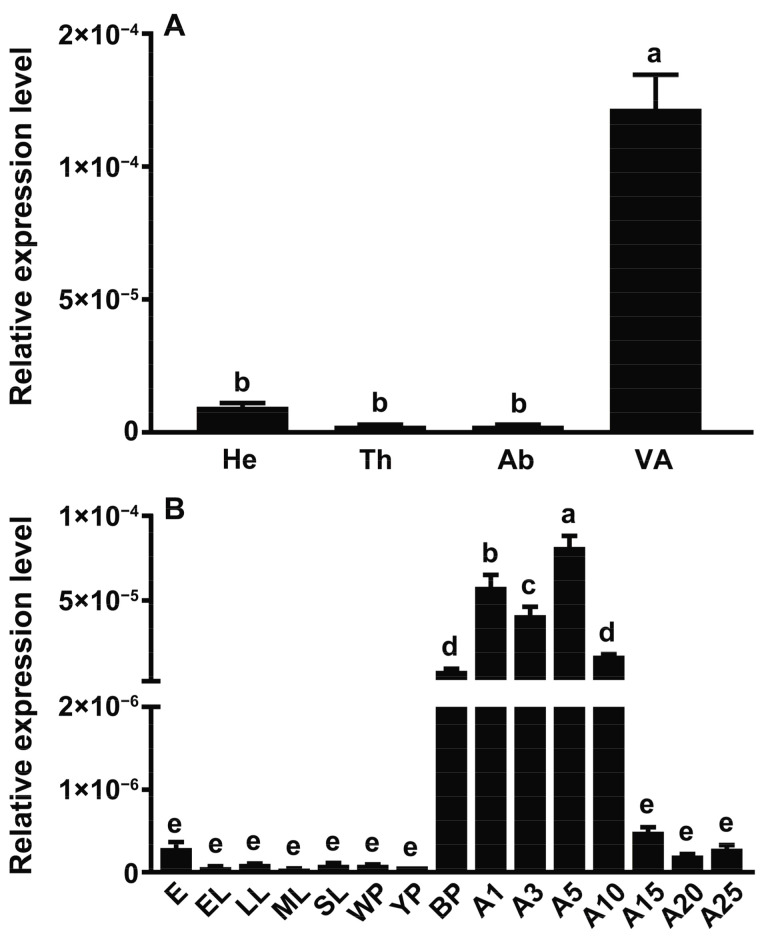
Gene expression profiles of SgVnDPPIV detected using quantitative real-time PCR. (**A**) Expression profiles of SgVnDPPIV gene in various tissues of female adults. He, head; Th, thorax; Ab, abdomen derived of venom apparatus; VA, venom apparatus. (**B**) Expression profiles of SgVnDPPIV gene at different developmental stages. E, egg; EL, early larvae; LL, late larvae; ML, mature larvae; SL, spinning mature larvae; WP, pupae in white cocoon; YP, pupae in yellow cocoon; BP, pupae in back cocoon; A1–25, adults after 1–25 days emergence. Different superscript letters indicate statistically significant differences among different samples.

According to the qPCR experimental results (Figure 3A), the SgVnDPPIV gene is most highly expressed in the venom apparatus. The transcriptional level of SgVnDPPIV in the venom apparatus is significantly higher than in abdomens deprived of the venom apparatus and other tissues. Its expressions are dynamic across life stage. *Scleroderma guani* expresses this venom gene at a very low or even undetectable level in eggs, larvae, and pupae in white and yellow cocoons (Figure 3B). However, its expression then significantly increases in pupae in black cocoons, and reaches to the highest expression level in newly emerged adults. The expression of the SgVnDPPIV gene remains at a high level in other detected adult stages, with an overall decreasing tendency.

### 2.3. Enzymatic Activity of SgVnDPPIV

**Figure 4:**
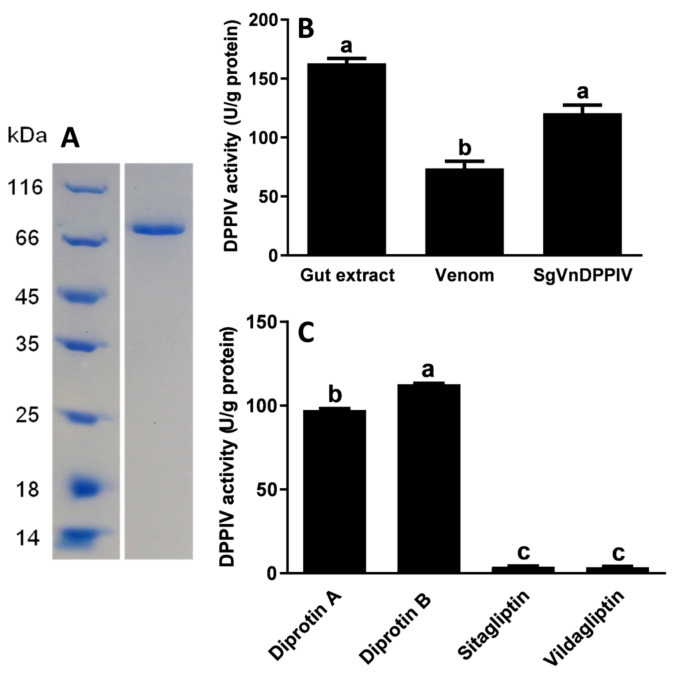
Enzymatic assay of recombinant SgVnDPPIV. (**A**) Expression of SgVnDPPIV in Sf9 cells using the baculovirus expression system. The recombinant SgVnDPPIV was purified and detected with Coomassie Brilliant Blue. (**B**) Enzymatic activity of gut extract of *Tenebrio molitor* larvae, venom of *Scleroderma guani*, and recombinant SgVnDPPIV. (**C**) Effect of inhibitors on the activity of recombinant SgVnDPPIV. Different superscript letters indicate statistically significant differences among different samples or treatments.

Western blot analysis showed that recombinant SgVnDPPIV was successfully produced in baculovirus-infected *Spodoptera frugiperda* Sf9 cells (Figure S2). The production was found in the cell medium and the soluble fraction of cell lysates. High-purity SgVnDPPIV was purified from concentrated cell culture supernatant (Figure 4A). Analyses of the enzymatic activity of SgVnDPPIV indicated that it has higher activity than that of crude venom from *S. guani* at their equivalent amounts (Figure 4B). Its activity towards the substrate of Ala-Pro-*p*-nitroanilide is comparable to the gut extract of *T. molitor* larvae. Vildagliptin and sitagliptin can efficiently inhibit the activity of SgVnDPPIV (Figure 4C). There are no significant differences between their inhibitory ability towards SgVnDPPIV. However, diprotin A and B have no evident inhibitory effect on SgVnDPPIV activity.

### 2.4. Functional Analysis of SgVnDPPIV

**Figure 5:**
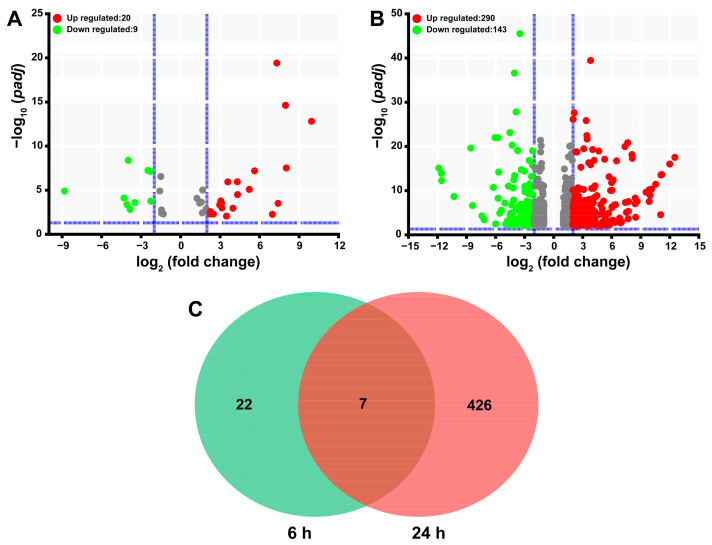
Differentially expressed genes in *Tenebrio molitor* pupae envenomated by SgVnDPPIV. (**A**) Volcano plot of differentially expressed genes identified 6 h post SgVnDPPIV injection. (**B**) Volcano plot of differentially expressed genes identified 24 h post SgVnDPPIV injection. (**C**) Venn diagram of differentially expressed genes identified 6 h and 24 h post SgVnDPPIV injection.

Transcriptomic sequencing led to the generation of 531555784 raw reads of SgVnDPPIV-injected pupae of *T. molitor* and their controls (Table S3). After adapter and low-quality nucleotide removal, the raw data were reduced to 79.31 G clean data. Clean reads from each sample were mapped to the genes encoding in the genome of *T. molitor* to calculate their transcript abundance, expressed in TPM (transcripts per million). Differential gene expression analysis revealed that a total of 455 genes are significantly differentially expressed in comparison with the control (Table S4), which only represents 0.99% of the total 45,780 genes encoding in the genome of *T. molitor*. Overall, injection with SgVnDPPIV, after 6 h and 24 h, influences the expression of 29 (20 upregulated and 9 downregulated) and 433 (290 upregulated and 143 downregulated) genes of *T. molitor* pupae, respectively (Figure 5A,B). In Figure 5C, the Venn diagram exhibits that the expression of only seven genes change at both time points upon injection with SgVnDPPIV.

**Figure 6:**
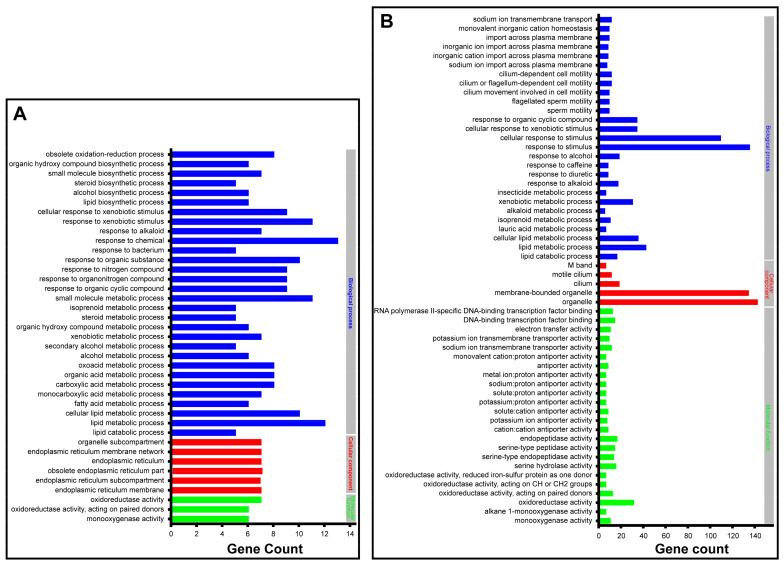
Gene ontology (GO) enrichment analysis at a broad level of differentially expressed genes in *Tenebrio molitor* pupae envenomated by SgVnDPPIV. (**A**) Enriched GO terms and differentially expressed gene number 6 h post SgVnDPPIV injection. (**B**) Enriched GO terms and differentially expressed gene number 24 h post SgVnDPPIV injection.

By utilizing GO enrichment analysis at level three, the significantly differentially expressed genes 6 h after injection with SgVnDPPIV were enriched in the categories of oxidoreductase activity (7), organelle subcompartments (7), small molecule metabolic processes (11), and response to chemical stimuli (13), respectively (Figure S3A). Those genes significantly differentially expressed 24 h after injection with SgVnDPPIV are enriched in the GO terms of M band (6), oxidoreductase activity (31), organelle (142), and cellular response to stimuli (109) (Figure S3B). Detailed examination of the functions of the enriched genes revealed that they are mostly related to detoxification, lipid synthesis and metabolism, response to stimuli of different compounds, such as xenobiotics, alkaloids, and alcohols, and ion exchange (Figure 6 and Table S5).

## 3. Discussion

The present study reports the cloning and sequencing of venom DPPIV cDNA from the ectoparasitoid, *S. guani* (SgVnDPPIV). Similar to mammalian DPPIV [3], SgVnDPPIV lacks a signal peptide and has a transmembrane domain, indicating that it is a transmembrane enzyme. In contrast, DPPIVs of *T. molitor* and some other insects are secreted enzymes [3]. Although most venom components are recognized as secreted proteins with a signal peptide, a few of them have been revealed to lack a predicted signal peptide [30]. Unlike most venom proteins, despite the fact that the characteristic signal peptide is present in DPPIV from the venom of the pit viper, *Gloydius blomhoffi brevicaudus*, it is not cleaved off during biosynthesis [31]. According to the phylogenetic result, SgVnDPPIV is grouped into a separate clade, and the other three non-venomous DPPIVs from *S. guani* are assigned into other separate groups, suggesting that their functions are different. A high gene expression level of SgVnDPPIV was observed in the venom apparatus of *S. guani*, and strong DPPIV activity was detected in its venom. According to the qPCR and enzymatic assay results, SgVnDPPIV is an abundant venom component of *S. guani*, which has been evidenced using the proteomic approach [29]. Abundant expression of the SgVnDPPIV gene occurs in pupae in black cocoons, and its expression levels are highly maintained at early adult stages, indicating that the transcription of this venom gene is correlated with the development of the venom apparatus, which is responsible for the production of venom components, and might be due to the biological rhythm of female wasps [32]. The three catalytic triads (Ser, Asp, and His) for serine peptidases are present in the deduced amino acid sequence of SgVnDPPIV [33]. Core substrate binding sites that are important for the activity of mammalian DPPIVs are highly conserved in the amino acid sequence of this venom protein [34,35]. These findings demonstrate that SgVnDPPIV is an active venomous enzyme. In agreement with this, the active recombinant SgVnDPPIV was produced in Sf9 cells for enzymatic and functional characterization.

The activity of SgVnDPPIV is sensitive to the inhibitors of vildagliptin and sitagliptin, but diprotin A and B have no obvious inhibitory effect on it. This is similar to DPPIVs of humans and *T. molitor*, which are not efficiently inhibited by diprotin A or B [3]. Vildagliptin and sitagliptin are the most effective inhibitors of human DPPIV [35]. Vildagliptin strongly inhibits *T. molitor* DPPIV, but its activity is not apparently influenced by sitagliptin [3]. These differences might be a result of the differences in the structures of the S1 and S2 subsites of DPPIVs from various species, which bind to different types of inhibitors with different modes of action.

DPPIV is an important enzyme used to degrade proline-containing peptides [2,9]. Recently, it has been found to participate in the hydrolysis of dietary gliadin, which is rich in proline residues, performing a digestive function in the larval gut of *T. molitor* [3]. As a venom component of the honey bee, hornet, and paper wasp, DPPIV was characterized as an allergen, and may be involved in processing peptidic toxins [12,13,14,15,16,17]. Venom of these wasps commonly causes allergic reactions among humans, and is used to defend their colonies from vertebrate predators; this is different from the venom of parasitoids, which is responsible for paralyzing hosts and manipulating their parasitized hosts for the survival and development of their progeny [21,36,37,38]. Additionally, peptides are often abundant in the venom of Aculeate bees and wasps, while they are rarely identified in the venom of parasitoids [39,40]. According to these differences in the venoms of Aculeate groups and parasitoids, DPPIV in the venom of parasitoids may act in biological processes other than allergic reactions or the maturation of proline-rich peptidic toxins, and might be shaped in response to both ecological and behavioral influences during the evolution of parasitoids.

In order to reveal the putative function of SgVnDPPIV, we investigated the influence of this venom protein on the expression of genes encoding in the genome of *T. molitor*. Only a very small portion of genes of *T. molitor* pupae changed their transcriptions after this beetle was envenomated with SgVnDPPIV. This is similar to venom Y, a major component of *Nasonia vitripennis* venom, in that the presence of this venom can only lead to changes of 1.8% of the total genes of its host, flesh fly *Sarcophaga bullata* [41]. The majority of them are detoxification, immunity, neural, and fat-body-specific genes [41]. Among the genes affected by SgVnDPPIV, many of them are involved in oxidation reduction, lipid synthesis and metabolism, response to stimuli, and ion exchange, indicating the corresponding function of SgVnDPPIV. These results pave the way towards a better understanding of the role of DPPIV in parasitoid venom; however, the mechanisms by which this is achieved remain to be unraveled.

## 4. Conclusions

Herein, venom DPPIV of *S. guani* (SgVnDPPIV) was molecularly characterized and functionally analyzed. The results indicate that this venom protein has conserved catalytic triads and substrate binding sites of mammalian DPPIV, exhibiting high enzymatic activity that can be inhibited by vildagliptin and sitagliptin. It can function by affecting the expression of hosts genes related to detoxification, lipid synthesis and metabolism, stimulus response, and ion exchange. These findings will facilitate the understanding of the biological role of DPPIV in the venom of parasitoids.

## 5. Materials and Methods

### 5.1. Insects

The parasitoid, *S. guani*, and its host, *T. molitor*, used in this study were derived from colonies maintained in our laboratory, as described by Zhu et al. [42]. In brief, newly emerged adult wasps were fed daily with fresh 20% honey solution in water at room temperature and 70% relative humidity under a 14 h light/10 h dark diurnal cycle. Pupae of *T. molitor* were supplied as hosts for parasitization by *S. guani*. These beetles were reared using wheat bran containing dry instant yeast as an artificial diet, and kept in the dark at about 55% relative humidity.

### 5.2. Gene Cloning and Sequence Analysis

The venom apparatus was dissected from about 150 female adults of *S. guani*. Total RNA was isolated from the venom apparatus using Trizol reagent (TaKaRa, Dalian, China). Its purity and concentration were measured using a NanoDrop ND 1000 spectrometer (PeqLab, Erlangen, Germany). Its quality was verified using 1.0% agarose gel electrophoresis. Aliquots of total RNA (5 µg) were used to synthesize the cDNA using a RevertAid First Strand cDNA Synthesis Kit (Thermo Fisher Scientific, Waltham, MA, USA) with the 3′-RACE CDS primer (5′-AAGCAGTGGTATCAACGCAGAGTAC(T)_30_-3′). The gene sequence of SgVnDPPIV was derived from the venom apparatus transcriptome of *S. guani* [29]. Based on this, the gene-specific forward primer (5′-GGAACAGTATCTGGACAGACTAAGGAAT-3′) was designed with Primer 5.0 (PREMIER Biosoft International, Palo Alto, CA, USA). This primer and the 3′-RACE CDS primer were used to clone the SgVnDPPIV gene. PCR (polymerase chain reaction) conditions were as follows: 3 min of pre-denaturation at 94 °C, 40 cycles of 30 s at 94 °C, 1 min at 60 °C, 2 min 30 s at 72 °C, and the final extension at 72 °C of 10 min. PCR product was detected using 1.0% agarose gel electrophoresis, and then sent to Biosune Biotechnology Limited Company (Shanghai, China) for sequencing. Signal peptide was predicted with SignalP 6.0 (https://services.healthtech.dtu.dk/service.php?SignalP-6.0 accessed on 20 October 2022) [43]. Genes encoding DPPIVs of various hymenopteran species were identified using the BlastP in TBtools v1.0987663 [44] from their genomes (Tables S1 and S2). Multiple sequence alignment of the amino acid sequences was performed with ClustalX 2.1 [45]. A phylogenetic tree was constructed using IQ-TREE 2 based on alignment with 1000 ultra-fast bootstrap replicates [46].

### 5.3. Quantitative Real-Time PCR

Selected tissues, including the venom apparatus, abdomens deprived of the venom apparatus, thoraxes, and heads from female adults and parasitoids from different developmental stages, including eggs, early larvae, late larvae, mature larvae, spinning mature larvae, pupae in white cocoon, pupae in yellow cocoon, pupae in black cocoon, and adults after 1–25 days emergence, were collected. Total RNA was isolated, and its purity and quality were verified as described above. Total RNA from these samples was used to synthesize cDNA with the PrimeScript RT Reagent Kit with gDNA Eraser (TaKaRa, Dalian, China). Gene-specific primers (5′-CGGATACAAGTGCAACGTCAG-3′ and 5′-TTGGTG GTCAAGTAGGAGTGG-3′) were designed using Primer 5.0 (PREMIER Biosoft International, Palo Alto, CA, USA) according to the corresponding gene sequence. The 18S ribosomal RNA gene was used as an endogenous control. Quantitative real-time PCR (qPCR) was performed using SYBR Premix Ex Taq II (TaKaRa, Dalian, China) to quantitatively evaluate the level of gene transcription in triplicate samples. Amplification reactions were 95 °C for 2 min followed by 45 cycles of 95 °C for 15 s and 58 or 60 °C for 30 s, finishing with elongation at 72 °C for 30 s. The 2^−ΔΔCT^ method was used to analyze the qPCR data [47].

### 5.4. Recombinant Production of Proteins

According to the sequence of the SgVnDPPIV gene described above, its cDNA containing *Nco I* and *Hind III* ends was synthesized via a PCR-based accurate synthesis protocol [48]. After validation by Sanger sequencing, it was cut with *Nco I* and *Hind III* and then cloned into the pFast-bac1 vector provided in the Bac-to-Bac^®^ Vector Kit (Thermo Fisher Scientific, Waltham, MA, USA), and the final vector was verified by sequencing. This was used to generate bacmid following the manufacture’s instruction. Generated bacmid was used to transfect the Sf9 cells cultivated in Sf-900 II SFM (Thermo Fisher Scientific, Waltham, MA, USA) media at a density of about 9 × 10^5^ cells/mL using Cellfectin II Reagent (Thermo Fisher Scientific, Waltham, MA, USA). Infected cells were maintained in Sf-900 II SFM at 27 °C and 100 rpm to amplify the baculovirus. At 72 h post infection, cells and medium were harvested to determine the expression of the recombinant protein using 10% SDS-PAGE. Recombinant SgVnDPPIV bacmid-derived baculoviruses were amplified in an additional round of infection for high levels of protein expression. The supernatant of cell medium was collected to purify the recombinant proteins by affinity separation with Ni-IDA -Sepharose CL-6B (Qiagen, Hilden, Germany) according to the manufacturer’s protocol. Bound protein fractions were eluted with 300 mM imidazole. Fractions containing the SgVnDPPIV were pooled and concentrated for subsequent 10% SDS-PAGE analysis and stained with Coomassie Brilliant Blue (TaKaRa, Dalian, China). Western blotting was performed using anti-His Tag mouse monoclonal antibodies (TaKaRa, Dalian, China). The concentration of purified proteins was measured by the Quick Start Bradford Protein Assay (Bio-Rad, Hercules, CA, USA). They were stored at −80 °C prior to use.

### 5.5. Enzyme Assay

The enzymatic activity of SgVnDPPIV was assayed with substrate Ala-Pro-*p*-nitroanilide (Sigma-Aldrich (Shanghai) Trading Co., Ltd., Shanghai, China) following the method described by Tereshchenkova et al. [3]. The substrate was initially dissolved in dimethylformamide, and its final reaction concentration was 0.25 mM. SgVnDPPIV (1 µg) was combined with 2.5 µL substrate. The freshly prepared PBS (phosphate-buffered saline) (0.1 M, PH 8.0) was added to the final volume of 200 µL. The mixture was incubated at 40 °C for 30 min. Its absorbance was measured periodically using an Infinite F50 Plus and Infinite F50 Robotic microplate reader (TECAN, Männedorf, Switzerland) at 405 nm for 60 min. Venom of *S. guani* and gut extract from the *T. molitor* larvae were used as the positive controls. The effects of the inhibitors, including vildagliptin, sitagliptin, diprotin A, and diprotin B (Sigma-Aldrich (Shanghai) Trading Co., Ltd., Shanghai, China), on the enzymatic activity of SgVnDPPIV were tested. The inhibitor concentration was 0.1 mM in the incubate assay with the SgVnDPPIV before its reaction with the substrate, as described above. All assays were performed in three independent biological replicates. The enzymatic activity was expressed in units (U).

### 5.6. Transcriptomic Sequencing and Analysis

SgVnDPPIV (0.25 µg in 2 µL PBS, PH 7.4) was microinjected into the pupae of *T. molitor* after larvae molt into pupae 24 h. Pupae injected with equal volume of PBS were set as controls. Subsequently, 6 h and 24 h after treatment, pupae were collected and grinded in liquid nitrogen with a mortar and pestle. Samples from at least five pupae were pooled together and set as one biological replicate. Three biological replicates were used for each treatment at each time point. Total RNA was extracted using Trizol reagent (TaKaRa, Dalian, China). Its quantity and quality were determined with the Agilent 2100 Bioanalyzer (Agilent Technologies, technologies, Santa Clara, CA, USA) and using 1% agarose gel electrogenesis. mRNA was isolated from the total RNA with oligo d(T) attached magnetic beads, and was used to construct the library using the TruSeq RNA Access Library Prep Kit (Illumina, San Diego, CA, USA). Libraries were sequenced on an Illumina HiSeq 2000 platform (Illumina, San Diego, CA, USA) and 150 bp paired-end reads were generated. Raw data were deposited into the National Center for Biotechnology Information (NCBI) Sequence Read Archive under BioProject accession PRJNA954966 and PRJNA955130. After quality control to remove residual adapters and low-quality nucleotides, clean reads were mapped to the gene repertoire encoded in the genome of *T. molitor* using the Kallisto Super Wrapper function in TBtools v1.0987663 [44]. This gene repertoire was generated by combining genes annotated in different genome versions of *T. molitor* sequenced by our laboratory [49] and two other groups [50,51], followed by reducing the redundant sequences using CD-HIT [52]. The number of mapped reads to each gene was used to measure the gene expression in TPM using DESeq2 [53]. The final set of differentially expressed genes was defined as those with a significant adjusted *p*-value below 0.05 and an absolute fold change above 1.5. Gene ontology (GO) enrichment for the differentially expressed genes with blast hits of an e-value ≤ 1 × 10^−5^ against the NCBI Nr protein database was performed with the GO Enrichment function in TBtools v1.0987663 [44,54]. Significantly enriched GO categories are defined with an adjusted *p*-value < 0.05.

### 5.7. Statistical Analysis

All data were analyzed by one-way analysis of variance (ANOVA) with the least significant difference (LSD) method using Data Processing System (DPS) software v20.00 [55]. Statistical significance was set at the *p* < 0.05 level.

## Data Availability

All the information is at this document.

## Supplementary Materials

The following supporting information can be downloaded at https://www.mdpi.com/article/10.3390/toxins15050311/s1. Figure S1: The nucleotide and deduced amino acid sequences of the SgVnDPPIV gene. Figure S2: Western blot analysis of the expression of recombinant SgVnDPPIV in Sf9 cells using the baculovirus expression system (1, cell supernatant; 2, cell lysis supernatant). Figure S3: Gene ontology (GO) enrichment analysis at three levels of differentially expressed genes in *Tenebrio molitor* pupae envenomated by SgVnDPPIV (A, enriched GO terms and differentially expressed gene number 6 h post SgVnDPPIV injection; B, enriched GO terms and differentially expressed gene number 24 h post SgVnDPPIV injection). Table S1: Number of DPPIV genes encoding in the genomes of various hymenopteran species. Table S2: Amino acid sequences of DPPIVs identified in the genomes of various hymenopteran species. Table S3: Statistics of the transcriptomic sequencing. Table S4: Detailed list of differentially expressed genes in *Tenebrio molitor* pupae envenomated by SgVnDPPIV. Table S5: Detailed results of the gene ontology (GO) enrichment analysis of the differentially expressed genes in *Tenebrio molitor* pupae envenomated by SgVnDPPIV.
