# Supplementary figures and images for: Molecular Characterization and Functional Analysis of the Dipeptidyl Peptidase IV from Venom of the Ectoparasitoid Scleroderma guani
Source: Toxins (Basel). 2023 Apr 27;15(5):311. doi: 10.3390/toxins15050311 (PMC10222045; doi:10.3390/toxins15050311)

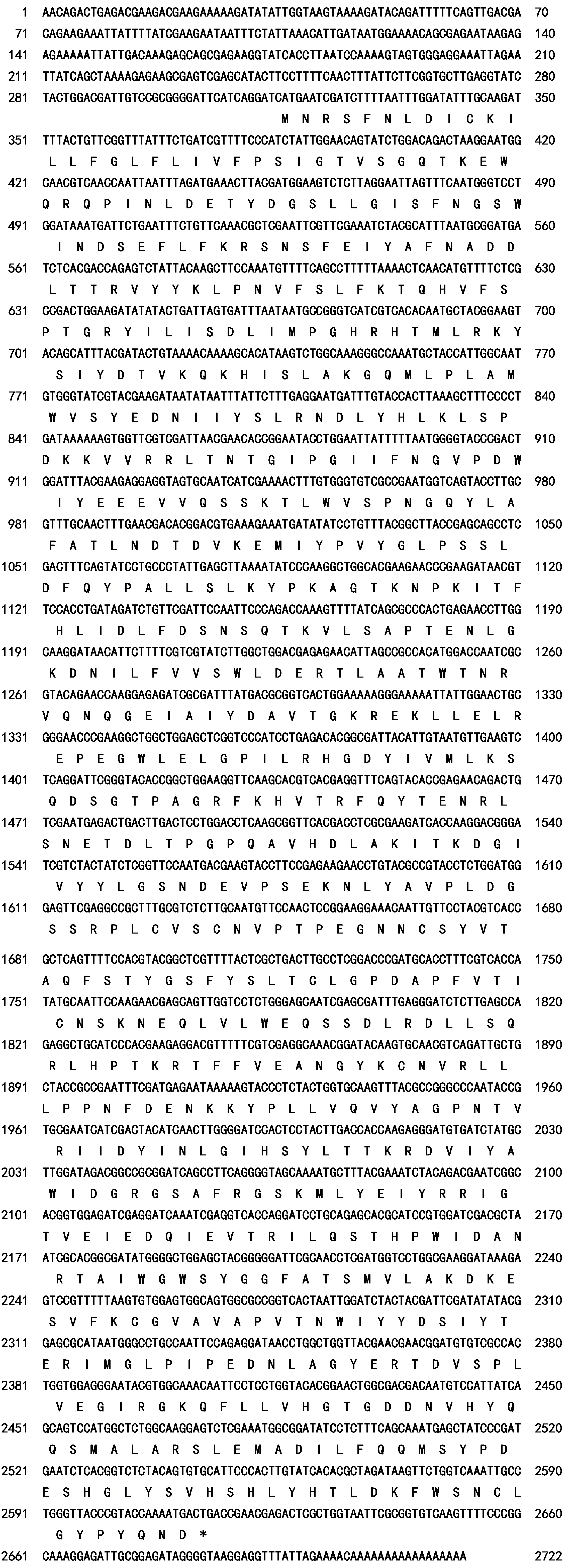

Supplement: Supplementary file 1 [file toxins-15-00311-s001.zip › Figure S1.tif]

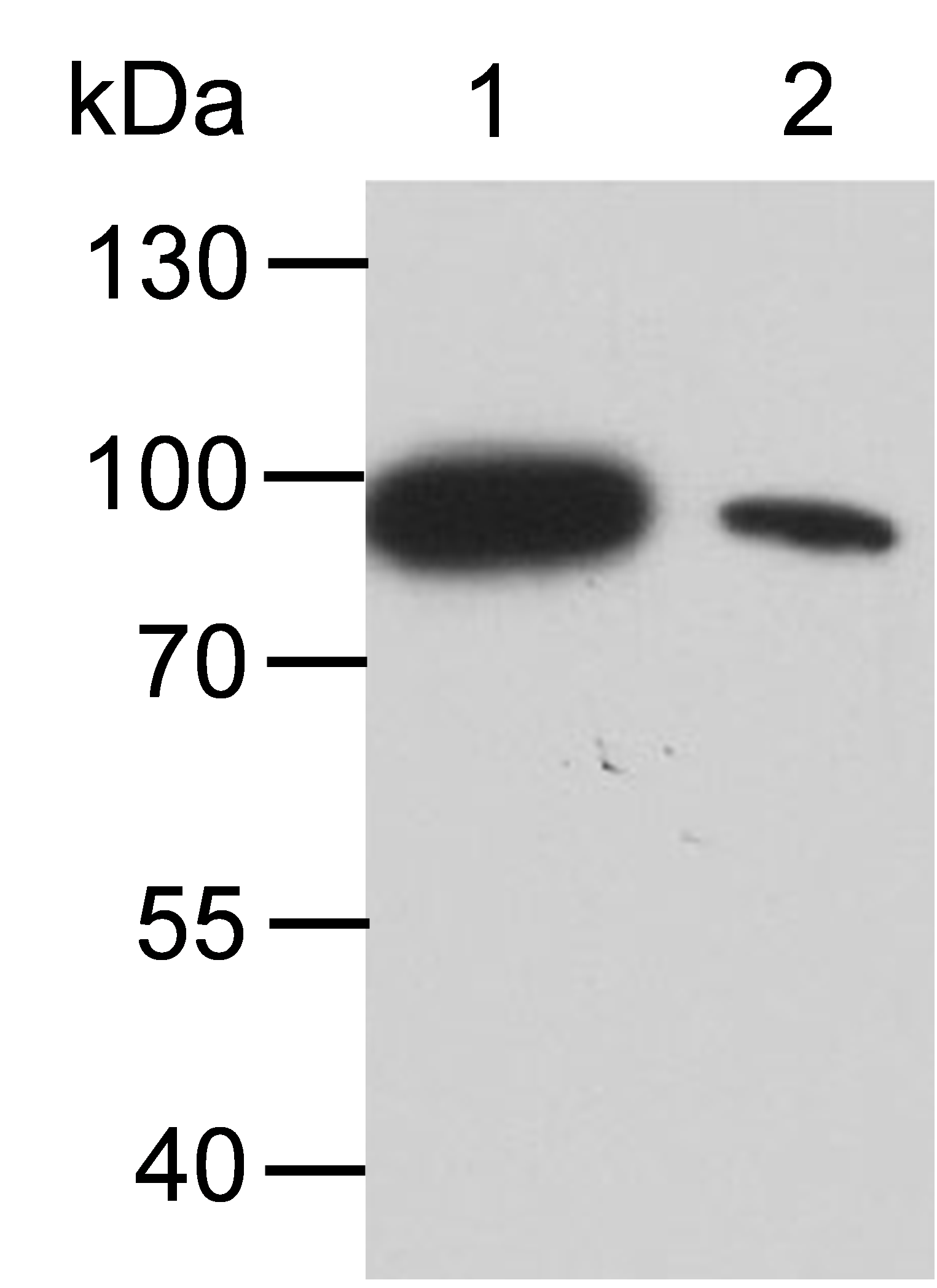

Supplement: Supplementary file 1 [file toxins-15-00311-s001.zip › Figure S2.tif]

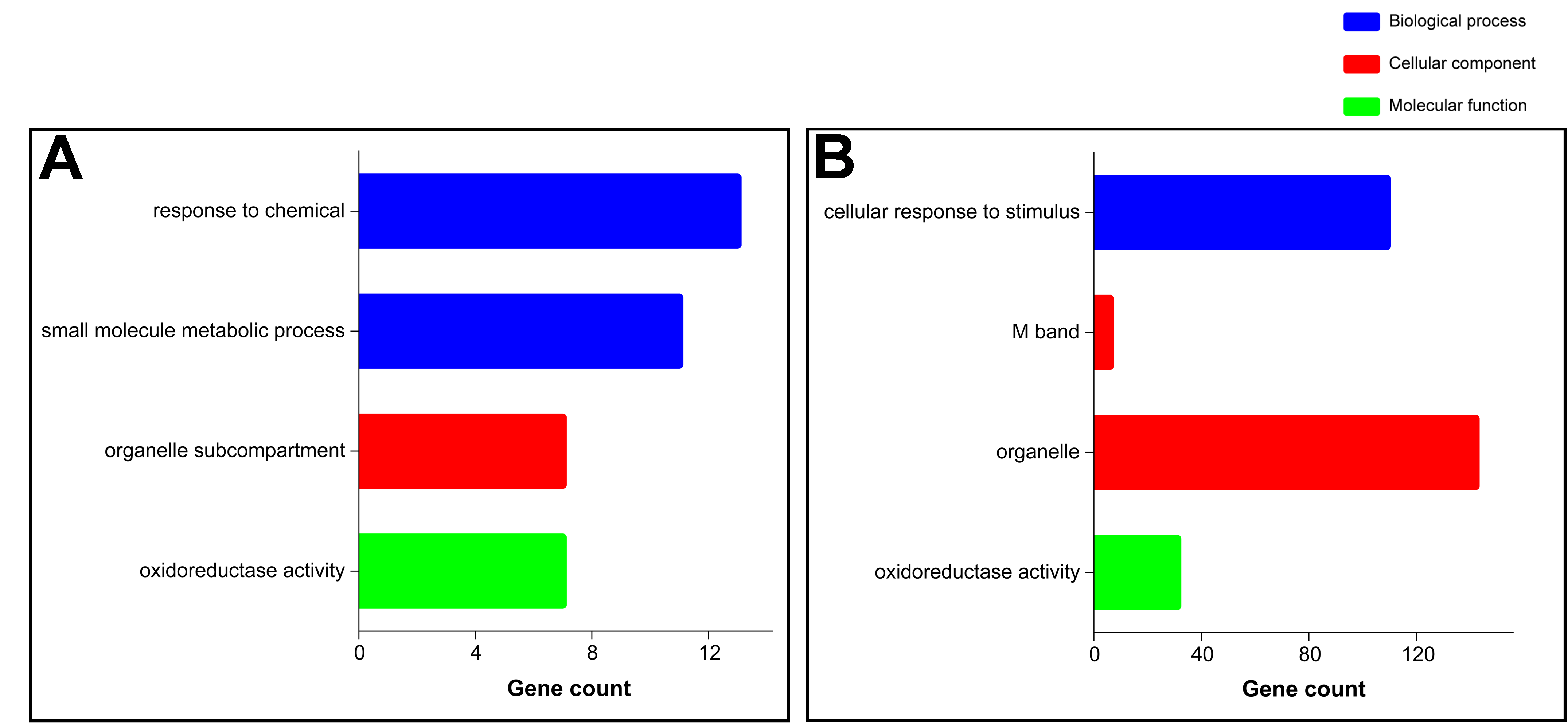

Supplement: Supplementary file 1 [file toxins-15-00311-s001.zip › Figure S3.tif]
